# Towards Oxidatively Stable Emulsions Containing Iron-Loaded Liposomes: The Key Role of Phospholipid-to-Iron Ratio

**DOI:** 10.3390/foods10061293

**Published:** 2021-06-04

**Authors:** Alime Cengiz, Karin Schroën, Claire Berton-Carabin

**Affiliations:** 1 Department of Food Engineering, Engineering Faculty, Ondokuz Mayis University, Samsun 55139, Turkey; alime.cengiz@wur.nl; 2 Food Process Engineering Group, Wageningen University, Bornse Weilanden 9, 6708WG Wageningen, The Netherlands; claire.berton-carabin@inrae.fr; 3 INRAE, UR1268 Biopolymères, Interactions, Assemblages, 44316 Nantes, France

**Keywords:** iron encapsulation, liposomes, oil-in-water emulsions, lipid oxidation

## Abstract

To encapsulate soluble iron, liposomes were prepared using unsaturated phospholipids (phosphatidylcholine from egg yolk), leading to high encapsulation efficiencies (82–99%). The iron concentration affected their oxidative stability: at 0.2 and 1 mM ferrous sulfate, the liposomes were stable, whereas at higher concentrations (10 and 48 mM), phospholipid oxidation was considerably higher. When applied in oil-in-water (O/W) emulsions, emulsions with liposomes containing low iron concentrations were much more stable to lipid oxidation than those added with liposomes containing higher iron concentrations, even though the overall iron concentration was similar (0.1 M). Iron-loaded liposomes thus have an antioxidant effect at high phospholipid-to-iron ratio, but act as pro-oxidants when this ratio is too low, most likely as a result of oxidation of the phospholipids themselves. This non-monotonic effect can be of crucial importance in the design of iron-fortified foods.

## 1. Introduction

Iron deficiency is a worldwide issue that may be mitigated by fortification of food products [1]. This sounds simple enough, but unfortunately soluble iron is also known to induce deleterious reactions such as protein and lipid oxidation, which damages health-promoting nutrients such as polyunsaturated fatty acids (PUFAs), gives undesired sensory effects, and possibly damages the nutritional quality of the products [2]. In order to fortify foods, it is thus required that soluble iron is prevented from inducing such negative side-effects, while still being bioavailable, which may be achieved by using encapsulation.

Liposomes are nano-size vesicles consisting of phospholipid bilayers that surround a core material containing an aqueous compartment, which makes them versatile carriers for bioactive materials [3]. They have unique characteristics, such as being self-sealing, natural, biodegradable, food-grade, and non-toxic [4], and thus appropriate for applications in food. They are known for their high encapsulation efficiency, simple production method, and high physical stability in products with high water activity [4]. They have been successfully used for encapsulation of various food ingredients such as enzymes [5], vitamins [6], food preservatives [7], and various sources of soluble iron [8,9,10,11], although some negative side effects have also been reported with regard to oxidation of the building blocks of the liposomes, i.e., unsaturated phospholipids [12].

Lecithin is a common food ingredient that is isolated from, e.g., egg or soybean, and is listed as generally-recognized-as-safe (GRAS). It is a mixture of phospholipids, which consist of a glycerol backbone with a phosphate-based group attached at the *sn*-3 position, and two fatty acids at the *sn*-1 and *sn*-2 positions [13]. Phospholipids are named after the chemical nature of the hydrophilic headgroup, for example phosphatidylcholine (PC), phosphatidylethanolamine (PE), phosphatidylserine (PS), and phosphatidylinositol (PI). The nature of the fatty acids depends on the biological source from which the lecithin is derived, and on possible post-treatments (e.g., hydrogenation); the main fatty acids encountered are palmitic, stearic, oleic, palmitoleic linoleic, and α-linolenic fatty acids [14].

Due to their amphiphilic structure, phospholipids can be involved in a range of colloidal structures, such as liposomes or other bi-layered structures (in aqueous phases), reverse micelles (in oil), or emulsion droplets (when adsorbed at the oil-water interface) [15]. Owing to the unsaturated fatty acids often present in their molecular structure, phospholipids are prone to lipid oxidation, which may physically alter the liposomes’ membrane, causing off-flavored components production to co-oxidize other nutrients present inside or outside the liposome compartments [13]. However, phospholipids can also act as antioxidants by chelating pro-oxidant metals [16,17,18], forming antioxidant Maillard reaction products [19], altering the location of primary antioxidants, regenerating primary antioxidants [14,20,21], or combinations thereof. Table S1 presents an overview of some effects reported in literature about the oxidative stability of liposomes, either investigated as such, or when incorporated in model food systems (e.g., food emulsions and fruit juice), as related to phospholipid type, metal ions, hydrating medium, antioxidants, and environmental conditions (i.e., temperature and pH). In general, when PC was combined with oil-soluble antioxidants (e.g., tocopherol, carotenoids, quercetin) this led to high stability against metal-catalyzed oxidation in liposomes. This is explained by the fact that lipophilic antioxidants spontaneously insert in phospholipid bilayers, i.e., at the location where phospholipid oxidation is initiated. This is in line with other sources stating that phenolipids, i.e., lipophilised antioxidants, are more efficient against liposome oxidation than hydrophilic ones, because they are able to cross or interfere with lipid membranes owing to their lipophilic tail [22,23]. In addition, such an insertion of amphiphilic antioxidants within phospholipid bilayers may make liposomes less permeable to aqueous solutes such as metals [22]. Table S1 remarks that in the most studies, metal ions were added as oxidation catalysts, not for fortification purposes, however, learnings can still be taken. It is clear that a knowledge gap needs to be bridged before PUFA-rich food emulsions can be fortified without negative effects.

Reducing the unsaturation degree of phospholipids by hydrogenation can be an efficient strategy to reduce oxidative issues; however, from a nutritional and consumer point of view, it is not preferred because this adds to the saturated fat content of the food and has been associated with the formation of trans double bonds, which is linked to health issues [13]. Furthermore, the use of synthetic antioxidants such as butylated hydroxytoluene (BHT), tert-butylhydroquinone (TBHQ), and ethylenediaminetetraacetic acid (EDTA) does not comply with current consumer choice for cleaner and simpler labels [13]. From this, it is clear that a possible strategy to produce PUFA-containing foods enriched in iron could be to deploy iron ions-loaded liposomes, providing that such structures can effectively circumvent undesired oxidative effects. This is a real challenge, and publications on this topic including fundamental understanding are still lacking. In the present study, we systematically investigate the effect of the phospholipid-to-iron ions ratio on the oxidative stability of liposome suspensions, which are next applied in surfactant-stabilized emulsions rich in PUFAs, such that the total iron concentration in the emulsion is kept constant, but its local concentration in the intra-liposome environment varies. The physical properties (particle size distribution and zeta-potential) and oxidative stability of both the liposomes and the emulsions were determined as function of time, and compared to appropriate reference systems, and we investigated the effect of the iron concentration on the oxidative stability of O/W emulsions containing iron-loaded liposomes.

## 2. Materials and Methods

### 2.1. Materials

Rapeseed oil purchased from a local supermarket was stripped by means of alumina to eliminate surface-active impurities and tocopherols following a previously established method [24]. L-α phosphatidylcholine (PC) from egg yolk (purity ~60%), cholesterol (purity ≥99%), diethyl ether, Tween 20, iron (II) sulfate heptahydrate, 3-(2-Pyridyl)-5,6-di(2-furyl)-1,2,4-triazine-5′,5′′-disulfonic acid disodium salt, sodium phosphate monobasic, sodium phosphate dibasic, and citric acid were obtained from Sigma-Aldrich (St. Louis, MO, USA). Different buffers were used for liposome and emulsion preparation: 10 mM phosphate buffer (pH 6.8), 10 mM citrate buffer (pH 6.0), and 10 mM phosphate-citrate buffer (pH 6.8). All chemicals were used were of analytical grade unless otherwise stated. Ultrapure water, obtained from a Millipore Milli-Q system (Darmstadt, Germany) was used for all the experiments.

### 2.2. Methods

The fatty acid composition and tocopherol content of lecithin were determined as described elsewhere [25,26]. The initial oxidative status of stripped rapeseed oil and lecithin was assessed by measuring their conjugated diene and hydroperoxide contents, para-anisidine value and thiobarbituric acid reactive substances (TBARS) content, as described in the following sections (corresponding values are reported in Table S2).

#### 2.2.1. Preparation of Liposomes

Liposome dispersions were prepared as described earlier [12] with slight modifications. In brief, 0.45 g PC and 0.018 g cholesterol were dissolved in 30 mL diethyl ether and mixed with 10 mL of the previously described buffers, or with water, which both may or may not contain ferrous sulfate. First, the mixture was sonicated with a probe sonicator (Microtip 3 mm, Branson Sonifier 250, Dietzenbach, Germany) for 10 min at a frequency of 20 kHz, power of 150 W, and amplitude of 40% in an ice bath to prevent heating up. After organic solvent removal by rotary evaporation (RC900, KNF, Oxfordshire, United Kingdom) at controlled reduced pressure at 45 °C, 25 mL buffer were added while gently vortexing to obtain the liposome suspension. The final iron concentration was 0.2, 1, 10, or 48 mM. The liposome suspensions were filtered (0.45 µm paper, Whatman, Sigma Aldrich, Zwijndrecht, The Netherlands). To determine the oxidative stability of iron-loaded liposomes, they were incubated at 40 °C up to 14 days. The liposomes used in emulsions were stored at 4 °C in a refrigerator for one day before adding them to emulsions. Samples were prepared twice independently, and each replicate was measured three times.

#### 2.2.2. Preparation of Emulsions

The aqueous phase of the emulsion consisted of phosphate buffer (10 mM, pH 6.8) and Tween 20 (1 wt.%). Tween 20 was dissolved in the aqueous phase the day before emulsion preparation and gently stirred overnight at room temperature. A coarse emulsion containing 20 wt.% stripped rapeseed oil was prepared using a rotor stator homogenizer (UltraTurrax T25 Basic Disperser with 25 mm diameter blade; Janke & Kunkel, IKA, Staufen, Germany) at 7000 rpm for 2 min, followed by 3 passes at 800 bar through a high pressure homogenizer (Microfluidizer M-110Y High Pressure Pneumatic equipped with a F12Y interaction chamber, Microfluidics, Los Angeles, CA, USA). Six hundred milliliters of emulsion were prepared for each batch. The cooling jacket of the homogenizer was filled with iced water to limit temperature rise during the emulsification process. The experiments were performed twice as independent duplicates.

#### 2.2.3. Physical Characterization of Liposomes and Emulsions

Encapsulation efficiency of liposomal iron: The iron content in the phase surrounding the liposomes was determined by the spectrophotometric ferene method [12]. Ferrous iron reacts with the chromogen ferene to form a blue chromophore, and the intensity of the color is proportional to the amount of iron present in solution. To a 0.5 mL sample, 1 mL of dissociation agent, which is a mixture of ascorbic acid (0.25 M), acetate buffer (1.4 M- pH 4.5) in 1:1 ratio (*v*/*v*), and 0.1 mL ferene solution (6 mM) were added, and the absorbance was measured at 593 nm. A calibration curve was used to determine the ferrous iron concentration.

To determine the concentration of free iron (dissolved in buffer/water and non-encapsulated) $\left[Fe_{f}\right]$, ferrous sulfate-loaded liposomal suspensions were placed in a centrifugal ultrafiltration filter (Amicon^®^ Ultra-0.5, 30 kDa MW, Merck, Darmstadt, Germany) and centrifuged at 14,000× *g* for 20 min at 20 °C. The filtrate was collected, and 500-fold diluted with ultrapure water. The total concentration of iron $\left[Fe_{T}\right]$ was determined by taking 1 mL liposomal ferrous sulfate suspension and adding it to 5 mL hydrochloric acid (37%), followed by burning at 100 °C for 20 min. The obtained samples were mixed with 1 mL HCl solution (10%) and diluted 100 times. The calculation of the encapsulation efficiency (EE) is shown in Equation (1):

(1) $$\mathrm{EE}\;(\%)=\left[\left(\left[Fe_{T}\right]-\left[Fe_{f}\right]\right)/\left[Fe_{T}\right]\right]*100,$$

Size distribution and zeta potential: The size distribution, polydispersity index (PDI), and zeta potential of the liposomes were measured with a dynamic light scattering instrument (Zetasizer Nano ZS, Malvern Instrument Ltd., Worcester, UK). This device was also used to determine the zeta potential of emulsion droplets. The refractive index of dispersant was set to 1.33 for water, to 1.47 for liposomal material, and to 1.45 for rapeseed oil. As was the case for liposomes, the emulsions were prepared twice independently, and from each emulsion three samples were 1000-fold diluted with ultrapure water or corresponding buffers prior to measurement, both immediately after preparation, and at the end of the incubation period. The zeta potential was expressed as a mean value in mV ± standard deviation.

The droplet size distribution of emulsions was determined using static light scattering (Mastersizer 2000, Malvern Instruments Ltd., Worcester, UK). The refractive indices of rapeseed oil (1.45) and water (1.33) were used as particle and dispersant indices, respectively. Five measurements were conducted per sample, and the data were reported as the DeBrouckere mean diameter (*d*_4,3_).

### 2.3. Chemical Stability of Liposomes and Iron-Fortified Emulsions

#### 2.3.1. Incubation Conditions

**Figure 1:**
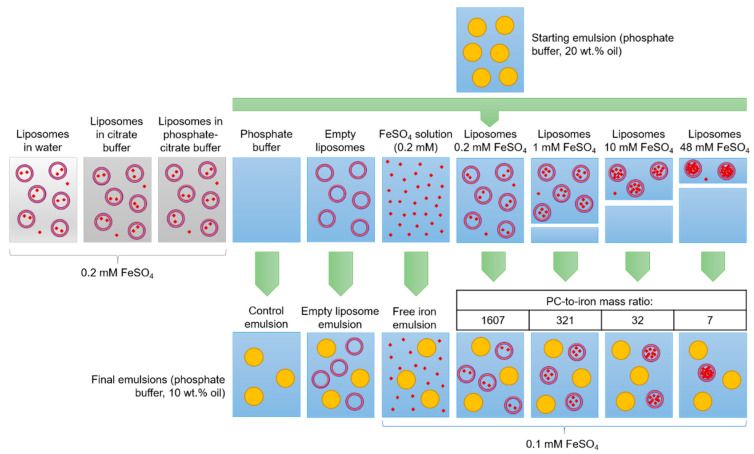
Schematic view of the preparation of emulsions that may or may not contain iron and/or liposomal material. The blue background corresponds to phosphate buffer, yellow spheres represent oil droplets, purple circles represent the liposomes, and small red dots represent soluble iron (not to scale). PC, phosphatidylcholine.

O/W emulsions containing 20 wt.% oil were mixed in a 1:1 ratio (*v*/*v*) with either liposome suspension or phosphate buffer that both may or may not contain iron, and gently vortexed to obtain homogenous systems. The combinations that were prepared are schematically shown in Figure 1. Seven emulsion systems were studied: control emulsions (no iron or empty liposomes added), emulsions with free iron (dissolved in buffer, non-encapsulated), and emulsions with iron-loaded liposomes that had been prepared with different iron concentrations (0.2 mM being the standard, the others were 1, 10, and 48 mM, respectively). The final emulsions all contained 0.1 mM iron, but the resulting amount of liposomal material (and hence the number of liposomes present in the continuous phase) was different. The final oil concentration in all emulsions was 10%. Samples were incubated at 40 °C on a rotating agitation device (5 rpm) for 7 to 14 days, with, for emulsion samples, ~40 g placed in 50-mL tubes, and for liposome suspension samples, ~20 g placed in 25-mL tubes.

#### 2.3.2. Lipid Oxidation Markers

Primary lipid oxidation products: The amount of hydroperoxides was measured using the method of Shantha and Decker [27]. Briefly, two stock solutions were prepared: for solution A, 0.8 g barium chloride was dissolved in 25 mL 0.4 M HCl, and for solution B, 7.5 g ammonium thiocyanate was dissolved in 25 mL ultrapure water: both solutions were stored cold and in the dark. To prepare the reagent, 0.14 M Fe_2_SO_4_ 7H_2_O was mixed with solution A in 1:1 (*v*/*v*) ratio and to obtain clear Fe (II) solution, barium chloride precipitate was centrifuged. Then, supernatant was mixed with solution B in 1:1 (*v*/*v*) ratio and the reagent was ready for use. 

To determine the hydroperoxide concentration, lipids were extracted from 0.3 g emulsion aliquots by mixing with 1.5 mL n-hexane:n-propanol (3:1, *v*/*v*). Next, 0.2 mL hexane phase, 2.8 mL methanol:1-buthanol (2:1, *v*/*v*) and 30 µL reagent were mixed. After 20 min incubation at room temperature, absorbance was determined at 510 nm against a blank containing all reagents except the sample using a spectrophotometer (DU 720 Beckman Coulter, Brea, CA, USA). The concentration expressed as mmol hydroperoxides per kg of oil was determined using a calibration curve obtained with a cumene hydroperoxide standard solution.

For stripped rapeseed oil and fresh PC, values of 0.05 and 11.23 mmol hydroperoxides per kg oil were found, respectively.

Secondary lipid oxidation products: The para-anisidine value (pAV), which is a global measurement of the formation of aldehydes, was determined by first mixing 0.3 g emulsion with 1.5 mL n-hexane:n-propanol mixture (3:1 *v*/*v*). The absorbance of the top hexane phase (*Ab*) was immediately measured at 350 nm, using pure hexane as blank. Then, 1 mL of this top hexane phase was added to 0.2 mL para-anisidine solution (2.5 M in acetic acid) and mixed. After 10 min the absorbance of this mixture (*As*) was measured using as blank pure hexane similarly mixed with the para-anisidine solution. The pAV was calculated as shown in Equation (2):

(2) pAV=(1.2·As−Ab)/m,

where *m* is the mass (g) of oil per mL hexane. For stripped rapeseed oil and PC, values of 0.02 and 0.51 (AU) were found, respectively.

Thiobarbituric acid reactive substances (TBARS) were determined according to a previously described procedure [28]. A trichloroacetic acid-thiobarbituric acid (TCA-TBA) solution (15 g TCA, 0.375 g TBA, 1.76 mL 12 N HCl and 82.9 mL of water) was mixed with butylated hydroxytoluene (2 wt.% solution in ethanol), in 100:3 (*v*/*v*) ratio. One milliliter emulsion was combined with 2 mL TBA solution, and the mixture was placed in a water bath at 75 °C for 30 min. The tubes were then cooled to room temperature for 10 min and then centrifuged (4000× *g*) for 15 min. The absorbance was measured at 532 nm. Concentrations of TBARS were calculated from a standard curve prepared with 1,1,3,3-tetraethoxypropane.

### 2.4. Experimental Design and Statistical Analysis

Statistical analysis was done using the SPSS software (version 18, PASW Statistics, Chicago, IL, USA). One-way analysis of variance (ANOVA) was conducted using six individual results from two independent repetitions and least significant differences were calculated at *p* < 0.05 applying Tukey’s post hoc test.

## 3. Results and Discussion

### 3.1. Iron-Loaded Liposomes

#### 3.1.1. Effect of Aqueous Phase Composition on Physical Properties 

The encapsulation efficiency of ferrous sulfate-loaded liposomes (0.2 mM iron) prepared in different buffers or ultrapure water was followed in time, as was the pH. The results are presented in Table 1. The aqueous phase had a significant effect on encapsulation efficiency; water and phosphate buffer led to high values of 99 and 96%, respectively, whereas the encapsulation efficiency measured for citrate and phosphate-citrate buffer was lower (82–87%). These results are different from those found by Ding et al. (2009) [9] who studied encapsulation of ferrous glycinate for which encapsulation efficiencies ranging from 75 to 30% were reported. The highest values were for the citrate-phosphate buffer, and the lowest for citrate buffer at pH 6.6, and the difference was explained by the excess level of citric acid that may dissociate ferrous glycinate [9]. It is good to point out that contrarily to our approach, in the work of Ding, the liposomes included Tween 80 and cholesterol, which are expected to modulate the liposome structure, stability and rigidity either by covering the phospholipid bilayer (Tween 80), or by cementing it (cholesterol) [9,29,30], or, if present in excess, by leading to leakage of the encapsulated material (of both compounds) [31].

The pH of the liposome suspensions in buffer did not change significantly over 14 days of incubation at 40 °C, but for water the pH dropped considerably. The starting pH of the iron-loaded liposome suspensions prepared in water was around 6.3 and decreased to 3.2 over 14 days incubation. When metal ions are present in water they form metal aquo complexes that are acidic owing, to the ionization of protons from the water ligands, which explains the eventual low pH of the solution [32].

The sizes of iron-loaded liposomes are given in Figures S1 and S2 and Table 2 (top section): they were rather similar in all tested media (no significant difference), ranging from 129, 139, 141, up to 154 nm in citrate, phosphate-citrate, phosphate buffers and water, respectively. The liposome size remained constant over 14 days incubation.

**Figure 2:**
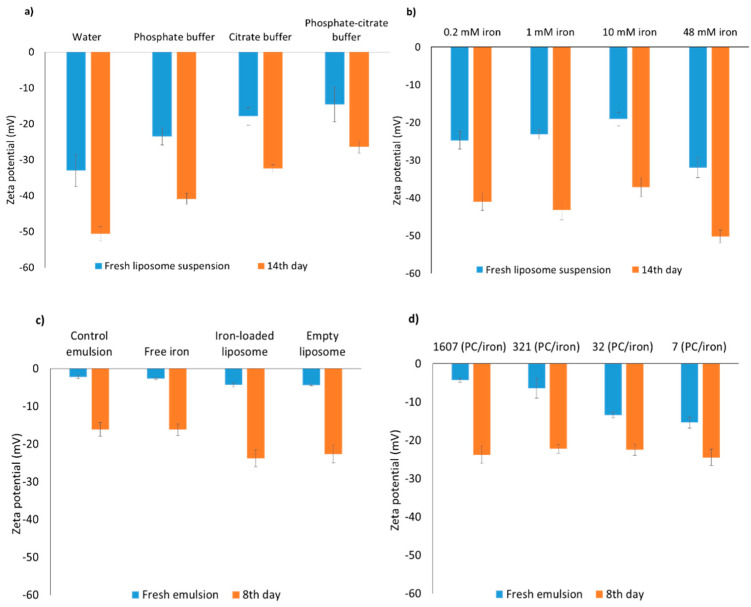
Zeta potential of fresh and incubated liposomes (40 °C) (**a**) in various buffers, with 0.2 mM iron; (**b**) in phosphate buffer, for various iron concentrations; and of (**c**) various reference emulsions, and (**d**) emulsions fortified with iron-loaded liposomes with various PC/iron ratio (*w*/*w*); please note that the bar corresponding to iron-loaded liposome in panel c, is the same as the first entry in d. All emulsions were incubated at 40 °C under slow rotative agitation; the total iron (FeSO_4_) concentration was always 0.1 mM. Error bars represent standard deviations (n = 4).

The zeta potential of all liposomes was negative (Figure 2a,b), and became more negative over 14 days incubation. A high net value of the zeta potential is linked to high repulsion forces between liposomes, which promotes high physical stability, as confirmed by the constant size in Table 2. The isoelectric point of PC is ~1, thus other phospholipids present in lecithin with higher IEP such as phosphatidylserine and phosphatidylglycerol could be responsible for the negative charge [12].

#### 3.1.2. Oxidative Stability of Iron-Loaded Liposomes

**Figure 3:**
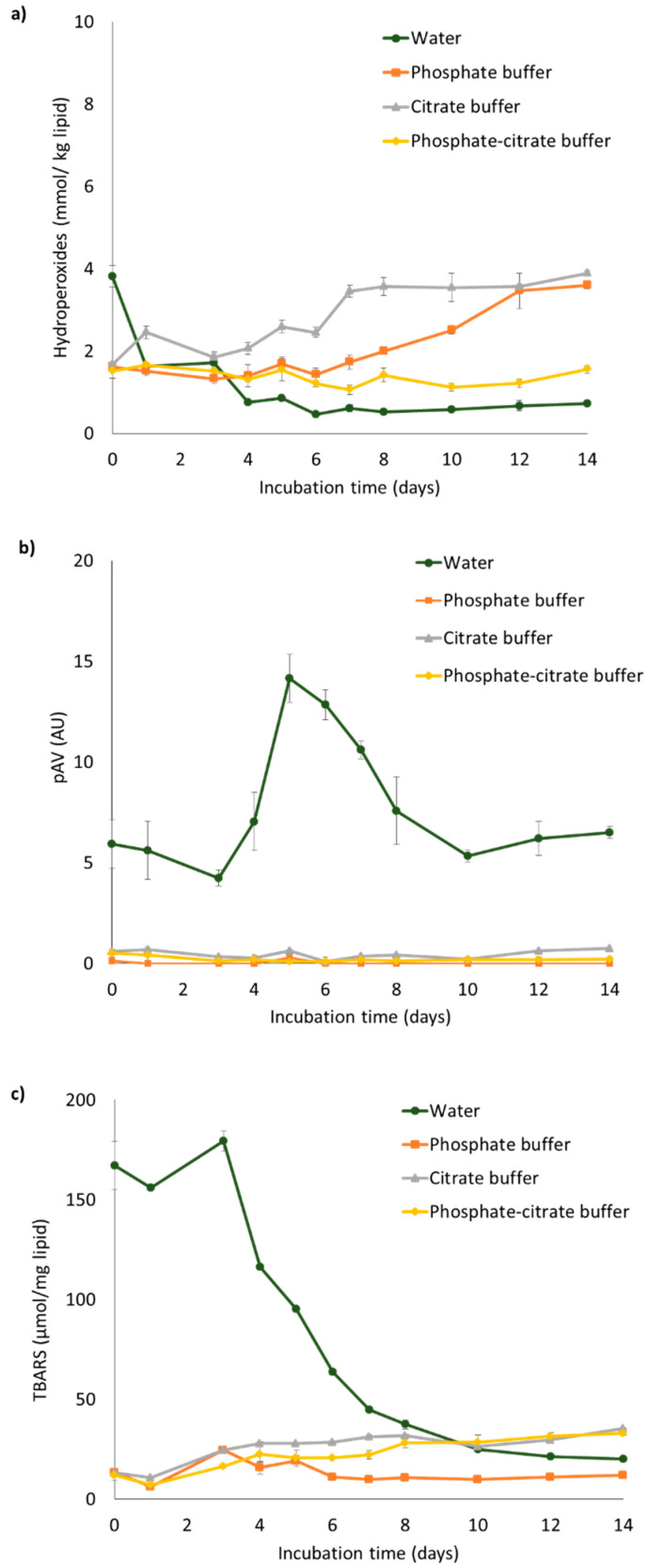
(**a**) Hydroperoxide concentration, (**b**) para-anisidine value (pAV), and (**c**) TBARS concentration in liposome suspensions prepared in water, phosphate, citrate, or phosphate-citrate buffer, with 0.2 mM ferrous sulfate. All liposome suspensions were incubated at 40 °C under slow rotative agitation. Error bars represent standard deviations (n = 4).

The effect of aqueous phase composition on the oxidative stability of liposomes containing 0.2 mM iron was evaluated by measuring primary (hydroperoxides) and secondary (para-anisidine value and TBARS) oxidation products for up to 14 days at 40 °C (Figure 3). Hydroperoxide concentrations remained under 4 mmol/kg oil for all liposome suspensions, whereas the pAV for liposomes in water dramatically increased to above 14 (AU) over five days and then decreased to 6.5 (AU) at the end of incubation, which also occurred for TBARS formation. pAV and TBARS concentrations remained low for the other samples over the timescale of the experiment, with the sample in phosphate buffer showing the lowest TBARS concentration at ~12 µmol/mg lipid at the end of the incubation. To summarize, iron-loaded liposomes showed a good stability to oxidation when present in buffer, whereas in water they were extremely unstable with a remarkably fast production of secondary lipid oxidation products (high pAV and TBARS). The fact that the latter markers show a subsequent decrease in time may be due to further decomposition of aldehydes into other products, such as alcohols and acids, which are not detected with the assays used in our study [32,33,34,35,36,37,38,39].

The pro-oxidant effect of ferrous iron is well documented [40], and more prominent at lower pH [18], which may explain the high oxidation rates measured for the liposomes in water for which the pH decreased strongly. Furthermore, it is known that both phosphate and citrate can dissociate in various negatively charged species that can interact with iron, or form complexes with iron (see Appendix A for more information), therewith rendering iron less active as a pro-oxidant. In addition, Goto et al.(1970) [41] found that spontaneous oxidation of ferrous iron can lead to various ferrous hydroxide complexes. Between pH 5 and 8, as is the case in our buffered liposome solutions, mainly Fe(OH)^2+^ is present, which reduces the actual iron concentration involved in oxidative reactions.

To wrap up, the buffer components chemically interact with iron via various routes. Overall, this decreases the pro-oxidant effect of iron by preventing it from producing reactive oxygen species (ROS; in particular ^•^OH radicals) via the Fenton reaction, and from reacting with lipid hydroperoxides. On top of this, phosphate ions can intervene in lipid oxidation through their free radical scavenging activity [41,42,43,44]. This combinatory effect of phosphate ions is interesting, since it may allow powerful mitigation of oxidative effects.

#### 3.1.3. Effect of Iron Concentration on Lipid Oxidation in Liposomes

**Figure 4:**
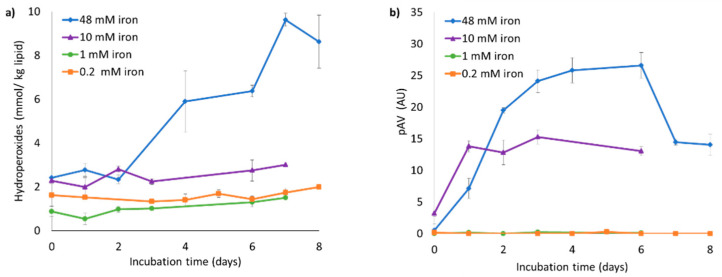
(**a**) Hydroperoxide concentration and (**b**) para-anisidine value (pAV) of iron-loaded liposome suspensions containing 0.2, 1, 10, or 48 mM iron, for a fixed PC concentration. All liposome suspensions were incubated at 40 °C under slow rotative agitation. Error bars represent standard deviations (n = 4).

The oxidative stability of liposome suspensions in phosphate buffer was further investigated using various ferrous sulfate concentrations, and hydroperoxide concentrations and pAV were measured over 14 days at 40 °C (Figure 4). Hydroperoxide concentrations for liposome suspensions containing 0.2 and 1 mM iron remained below 3 mmol/kg oil, whereas higher iron concentrations (10 and 48 mM) led to the formation of higher amounts of primary and secondary lipid oxidation products, as can be clearly seen through the pAV. Liposomal phospholipids in the phosphate buffer therefore seem to resist (or possibly delay) iron-catalyzed oxidation only until a certain iron concentration, above which they oxidize rapidly.

When liposomes are prepared by sonication, generation of traces of lipid peroxides (LOOH) is inevitable [45]. Besides, iron can bind to the membrane of the phosphatidylcholine-based liposomes, thus promoting phospholipid oxidation. District et al.[46] suggested that during the period when oxidation product concentrations are very low (latent period or lag phase), addition of ferrous iron (Fe^2+^) initiates lipid peroxidation by decomposing preformed hydroperoxides (LOOH) to peroxyl radicals (LOO*) that propagate oxidation. In the same work, the effect of the iron-to-lipid ratio on liposome oxidation was investigated, and it was found that when [Fe^2+^/LH] reached a critical value (40 µM Fe^2+^ in the continuous phase in their case), LOOH concentration increased rapidly and peroxidation started.

The ability of the polar headgroup of phospholipids, such as PC, to protect their fatty acid chains against oxidation has been reported in literature (Table S1, Supporting information). Accordingly, the antioxidant effect of PC and of other phospholipids with an anionic polar headgroup is linked to their ability to bind positively charged metals such as iron [18,47,48,49], thus reducing the ability of the metal ions to decompose lipid hydroperoxides to secondary oxidation products. Quite logically, the oxidative susceptibility of phospholipids has been reported to vary because of differences in the degree of unsaturation [13], although Yoshida et al. (1991) observed that both saturated and unsaturated PS protected egg yolk PC-based liposomes from oxidation by binding free iron to their anionic polar headgroup. Furthermore, a few studies reported that the fatty acids present in phospholipids oxidized faster when the headgroup was PE, compared to PC [17,50], which was found both in liposome and emulsion systems. This may be due to the sensitivity to oxidation of the ethanolamine group itself, which has been shown to occur concomitantly with the oxidation of the unsaturated fatty acid chains [50].

From this short literature impression, it is clear that the mechanisms involved in the oxidation of phospholipid-based matrices have been far from agreed on, and depend on the chemical nature of the components used, as well as on the colloidal structures in which phospholipids are involved. The systematic approach that we use here, first investigating the effect of iron concentration in liposomes, from which it is clear that the iron-to-phospholipid concentration is of pivotal importance, and next using this knowledge to design iron-fortified emulsions, can thus help unravel some of the hierarchical and cascaded effects involved. 

### 3.2. Iron-Fortified Emulsions

Various emulsions were incubated in oxidative conditions, amongst which there are various reference systems (Figure 1): a control emulsion without iron and liposomes, an emulsion with free iron, and an emulsion with empty liposomes. The purpose of these experiments was to elucidate the effect of PC-to-iron ratio while keeping the total iron concentration constant at 0.1 mM. Both physical (size distribution and zeta potential) and oxidative (hydroperoxide concentration and pAV) stability-related parameters were determined. 

### 3.2.1. Physical Properties of Iron-Fortified Emulsions

Table 2 (bottom section) shows that the droplet size (*d*_4,3_) was very similar for all emulsions, around 150 nm on average, and did not change during incubation. The zeta potential of all emulsions was negative even though a non-ionic surfactant was used, which is in agreement with results from others [12,18]; the possible reason being the preferential adsorption of OH^−^ onto the emulsion droplet surface [51], or binding of phosphate ions [12]. After 8 days of incubation, the charge of the emulsion droplets became more negative, yet still less negative than previously found for liposomes. This decrease in zeta potential in emulsions over incubation (Figure 2c,d) could be due to minor hydrolysis of the oil’s triglycerides into free fatty acids, which have affinity for the droplet surface, as reported previously [52].

#### 3.2.2. Oxidative Stability of Iron-Fortified Emulsions

**Figure 5:**
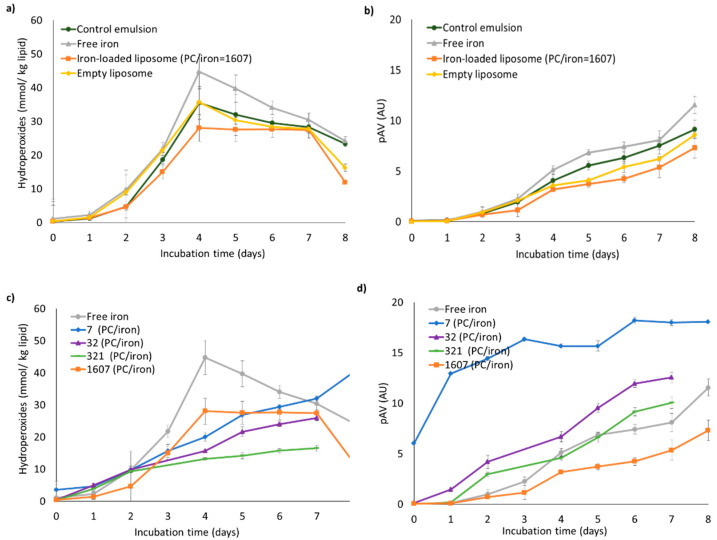
(**a**,**c**) Hydroperoxide concentration and (**b**,**d**) para-anisidine values (pAV). Top panels: control emulsion, emulsion with empty liposome, emulsion with iron-loaded liposome, and emulsion with free iron; bottom panels: emulsion with free iron and emulsions containing the following PC-to-iron ratios: 7, 32, 321, or 1607. All emulsions were incubated at 40 °C under slow rotative agitation. in all iron-containing emulsions the concentration was 0.10 mM. Error bars represent standard deviations (n = 4).

Effect of encapsulation of iron on its pro-oxidant activity in emulsions. Lipid oxidation in emulsions was evaluated based on hydroperoxide concentration and pAV for up to seven days at 40 °C (Figure 5a,b). During the first two days, in all emulsions the hydroperoxide concentration remained below 10 mmol/kg oil. After the fourth day, maximum values of 28–44 mmol/kg oil were reached, which decreased to 12–24 mmol/kg oil at the end of incubation. The pAV increased in all emulsions, eventually reaching 7–11 in emulsions containing free iron or iron-loaded liposomes; the PC-based liposomes as such did not influence lipid oxidation, as is evident from the empty liposome-emulsion results. Free iron led to a more pronounced lipid oxidation in the emulsion compared to iron entrapped in liposomes (for a PC-to-iron mass ratio of 1607, i.e., when the initial liposome suspension was prepared with 0.2 mM iron), which shows that the physical location of iron, and in particular, its segregation from the oil droplet substrate, can mitigate its overall pro-oxidant activity. In Figure 3, we already showed that at low iron concentration (0.2 mM), the liposomal material was stable to oxidation. Furthermore, the polar headgroups of PC may have bound iron, as described earlier by Cardenia et al. (2011).

When liposomes were initially prepared with higher iron concentrations (i.e., the iron concentration in the intra-liposome environment was high, and thus the PC-to-iron ratio low), the liposomes themselves were prone to oxidation, even in phosphate buffer (Figure 4). Whether this effect also spills over to the emulsion droplets was investigated next (Figure 5c,d).

Effect of PC-to-iron ratio: Iron-loaded liposome suspensions were prepared with various iron concentrations, and at a fixed amount of PC, and added to emulsions to reach 0.1 mM total iron concentration in all fortified emulsions (Figure 1). Primary and secondary lipid oxidation products were measured (Figure 5c,d). Hydroperoxide concentrations and pAV increased over the incubation period and were largely affected by the PC-to-iron ratio. The high lipid oxidation at low PC-to-iron mass ratios (7 and 32), i.e., when the local intra-liposome concentration of iron was very high, can be explained by the early formation of lipid radicals and hydroperoxides within the liposome membrane, in accordance with the data reported in Figure 4, and also in previous work [12], which could be reactive intermediates that would contribute to propagating lipid oxidation. It is remarkable that at these low PC-to-iron ratios, lipid oxidation in the emulsions was largely accelerated compared to the reference emulsion (free iron) with a similar total concentration of iron. This indicates that under such conditions, local accumulation within the liposomes in fact potentiates the pro-oxidant effect of iron, most likely by creating highly reactive “hot spots” within the emulsion, which could locally generate high concentrations of lipid oxidation products. This is in line with recent theories considering lipid oxidation as a spatially heterogeneous reaction [53,54,55,56].

At high PC-to-iron ratios, as also discussed earlier, lipid oxidation was clearly slower than at low ratios, which is especially visible in the pAV values, i.e., with regard to the formation of secondary oxidation products. At high ratios, i.e., when the local intra-liposome concentration of iron was low, lipid oxidation in emulsions proceeded slower than in the reference emulsion that contained non-encapsulated iron, showing that the antioxidant potential of these encapsulates can be tuned and even reverted depending on the PC-to-iron ratio, while keeping the total iron concentration constant. These are very important insights that need to be generalized to achieve iron-fortified emulsion products that do not suffer from lipid oxidation-related alterations.

## 4. Conclusions

Encapsulation of soluble iron in liposomes is possible at high encapsulation efficiency, and by tuning the ratio between phospholipids and iron, it is also possible to mitigate negative oxidation effects towards the liposomes themselves, and the surrounding lipid-containing matrix. This is an important step towards the design of PUFA-rich foods that are fortified in soluble iron.

## Figures and Tables

**Table 1:** Encapsulation efficiency (%) and pH of liposome suspensions prepared in various aqueous phases with 0.2 mM FeSO_4_. Values are the average ± standard deviation of three measurements done on two independently prepared batches.

Buffer	Set pH	Encapsulation Efficiency (%)	Actual pH	
			Fresh	After 14 Days at 40 °C
Water	-	99.4 ± 0.19	6.3 ± 0.11	3.2 ± 0.02
Phosphate buffer (10 mM)	6.8	95.9 ± 1.37	6.7 ± 0.08	6.4 ± 0.05
Citrate buffer (10 mM)	6.0	86.6 ± 0.18	6.0 ± 0.13	5.9 ± 0.04
Phosphate-citrate buffer (10 mM)	6.8	82.1 ± 4.46	6.8 ± 0.12	6.8 ± 0.11

**Table 2:** Size of iron-loaded liposomes, and of O/W emulsion droplets. Reported values are the average ± standard deviation of the nine measurements done for two independently prepared batches.

		Sample	Average Size (nm) *	
			Fresh	14th/8th Day **
Liposomes		Water	154 ± 0.5	153 ± 0.6
		Phosphate buffer	141 ± 0.2	141 ± 0.2
		Citrate buffer	129 ± 0.1	128 ± 0.5
		Phosphate-citrate buffer	139 ± 0.1	137 ± 0.2
Emulsions in phosphate buffer	Reference emulsions	Control	147 ± 0.1	148 ± 0.1
		+ Empty liposomes	146 ± 0.1	147 ± 0.1
		+ Free iron	144 ± 0.1	145 ± 0.1
	Emulsions containing iron-loaded liposomes	0.2 mM ***	148 ± 0.1	147 ± 0.1
		1 mM ***	146 ± 0.1	145 ± 0.1
		10 mM ***	146 ± 0.1	147 ± 0.2
		48 mM ***	148 ± 0.1	147 ± 0.1

* For liposomes, size was determined by dynamic light scattering and is reported as hydrodynamic diameter; for emulsion droplets, size was determined by static light scattering and is reported as *d*_4,3_. ** After 14 days incubation at 40 °C for liposomes, and after 8 days incubation at 40 °C for emulsions. *** Iron concentrations in the initial liposome suspensions used to fortify the emulsions; in all emulsions, the final iron concentration was 0.1 mM.

## Data Availability

Data sharing not applicable.

## Supplementary Materials

The following are available online at https://www.mdpi.com/article/10.3390/foods10061293/s1, Figure S1: Particle size distribution in iron-loaded liposomes, Figure S2: Particle size distribution in (a) the reference emulsion; (b) emulsions containing iron-loaded liposomes, Table S1: Overview of studies pertaining to lipid oxidation in liposome suspensions and liposome-containing systems, with a focus on (but not limiting to) those conducted in the presence of added metal ions. Table S2: Fatty acid composition (µg/g) of phosphatidylcholine (PC). References [8,12,18,37] are cited in the supplementary materials.

## Appendix A. Iron Complexation in Buffers

Buffers such as phosphate and citrate anions chelate iron cations, and in this way decrease their prooxidant potential [33,34,35,36]. At high pH, citrate has a negative charge and is able to complex iron, and an equilibrium is established between bound and unbound iron.

Citric acid and phosphate both have three pKa values (3.13, 4.76, and 6.4 for citric acid, and 2.15, 7.2, and 12.33 for phosphate). The various charged from can be calculated on classic Henderson-Hesselbach equations. At pH 6.8, the monohydrogenocitrate (CitH^2−^) majority, and the percentage of (CitH^2−^) of total citrate (CitH^2−^ + Cit^3−^) can be calculated as follows (adopted from Daoud et al. (2021) [33]):

(A1) $$\mathrm{pH}=\mathrm{pKa}+\log\frac{\mathrm{base}}{\mathrm{acid}}=\mathrm{pKa}+\log\frac{\left[\mathrm{Cit}H^{2-}\right]}{\left[Cit^{3\;-\;}\right]},$$

(A2) $$\frac{\left[CitH^{2-}\right]}{\left[Cit^{3-}\right]}=10^{\left(pKa‒pH\right)}\;=10^{\left(6.4‒6.0\right)}=2.52,$$

(A3) $$\frac{\left[CitH^{2-}\right]}{\left[CitH^{2-}\right]+\left[Cit^{3-}\right]}=\frac{2.52\left[Cit^{3-}\right]}{2.52\left[Cit^{3-}]+[Cit^{3-}\right]}=\frac{2.52}{1+2.52}=0.712.$$

At the buffer pH’s used in our work, citric acid is mostly in charged forms, either as monohydrogenocitrate (CitH^2−^) or totally deprotonated (Cit^3−^).

The distribution of phosphate species can be evaluated using the same strategy. At pH 6.8, phosphate is mainly presented as dihydrogenophosphate H_2_PO_4_^−^ (71%), and monohydrogenophosphate HPO_4_^2−^ (29%).

Whether stemming from citrate or phosphate, the ionic species present in the buffered systems we used can act as counter-ions or take part in the formation of hypervalent iron-oxygen complexes.

The complexation of ferrous ions with citrate and phosphate buffer is presented in the reactions (Equations (A4) and (A5)) and (Equations (A6) and (A7)), respectively [34,38]:

Fe^2+^ + HCit^2−^ → FeHCit, (A4)

Fe^2+^ + Cit^3−^ → FeCit^−,^ (A5)

Fe^2+^ + H_2_PO_4_^−^ → FeH_2_PO_4_^+,^ (A6)

Fe^2+^ + HPO_4_^2−^ → FeHPO_4._ (A7)
