# Supplementary material for: Towards Oxidatively Stable Emulsions Containing Iron-Loaded Liposomes: The Key Role of Phospholipid-to-Iron Ratio
Source: Foods. 2021 Jun 4;10(6):1293. doi: 10.3390/foods10061293 (PMC8230301; doi:10.3390/foods10061293)
Supplement: Supplementary file 1 [file foods-10-01293-s001.zip › foods-1170892-supplementary.pdf]

Supplementary Materials:

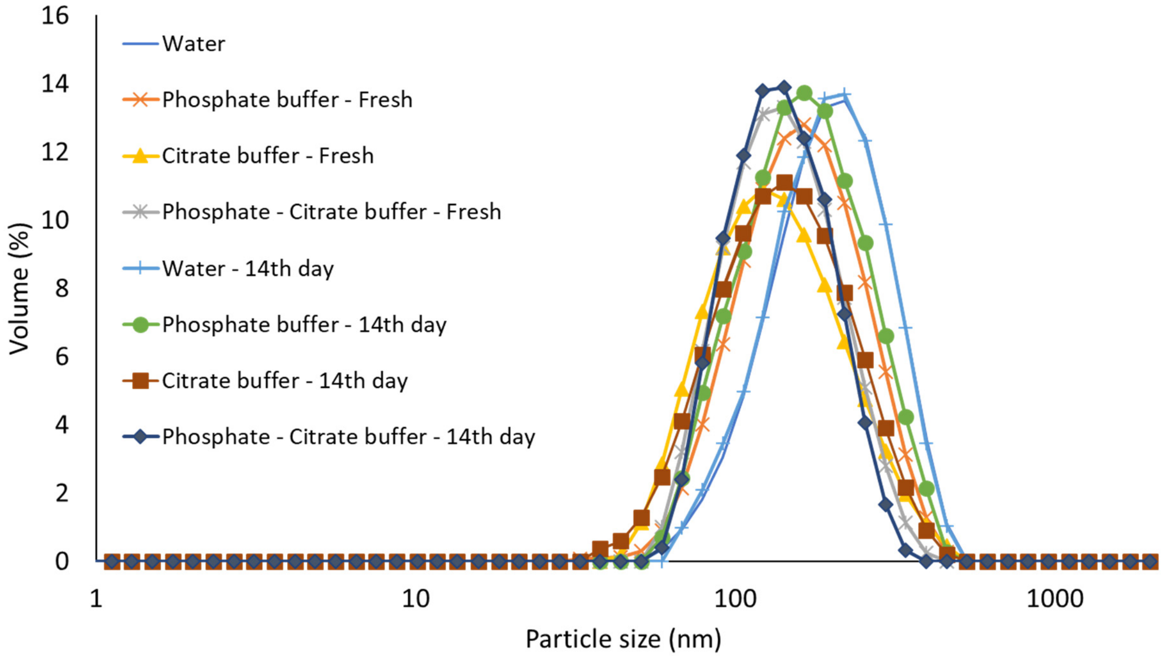

Figure S1. Particle size distribution in iron-loaded liposomes

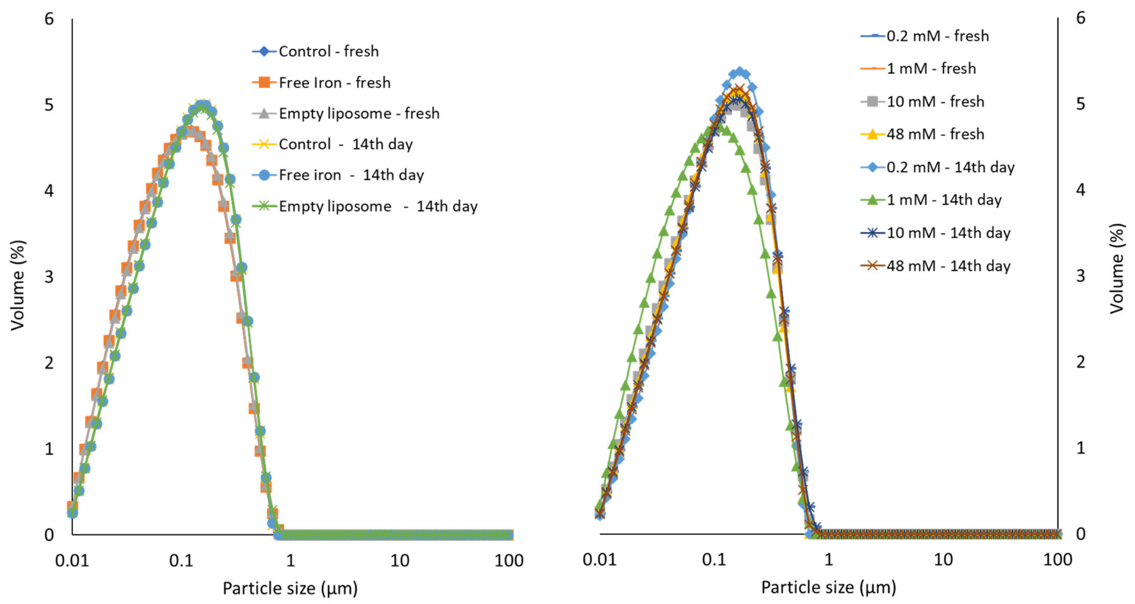

Figure S2. Particle size distribution of iron-loaded liposome added/not added emulsions: (**a**) reference emulsion; (**b**) emulsions containing iron-loaded liposomes

Table S1. Overview of studies pertaining to lipid oxidation in liposome suspensions and liposome-containing systems, with a focus on (but not limiting to) those conducted in the presence of added metal ions.

Reference	Phospholipid type	Aqueous medium	Other components	Oxidation prevention	Factors	Pro-oxidant	Key findings
[57]	Cod PC	5mM MES buffer (pH 5.5)	Cholesterol	-	NaCl, pH, iron, temperature, lipid concentration	Ferrous sulfate, ferrous chloride (2-20 μm)	Decrease in NaCl, increase in temperature and pH (4-5) and phospholipid concentration increase oxidation.
[58]	Egg PC, DPPC	10mM Tris–HCl buffer (pH 7.4)	Triphenylphosphine (TPP)	SOD, catalase, sodium benzoate, mannitol, dimethyl thiourea, EDTA	Antioxidants, iron concentration	Ferrous sulfate, ferric chloride (100 μM)	No other antioxidant but EDTA inhibited oxidation. Fe^{3+} stimulates Fe^{2+}-induced oxidation by accelerating ROS production. LOOH in PC is not directly involved in oxidation.
[8]	Pro-Lipo S (Soy PC), Emultop	Water	-	Ascorbic acid	Type of PCs	Ferrous sulfate (8-22 μM)	Emultop liposomes oxidatively more stable than PC liposomes because of high concentration of unsaturated phospholipids.
[59]	Soy PC, Dimyristoyl PC (14:0)	0.1 M sodium chloride solution	-	α-tocopherol	Iron concentration, natural and artificial PCs	Ferrous sulfate, ferric chloride (72-700 μM)	Iron in ferrous form: α-tocopherol reduced oxidation. Iron in ferric form: presence of α-tocopherol in liposomal membrane acted as pro-oxidant.
[60]	Egg PC	50 mM acetate buffer (pH 5.6)	-	DTPA, DFO	Concentration of metMb, presence of ferric chloride, antioxidants	Metmyoglobin (metMb), Ferric chloride (5 ppm)	Increasing MetMb conc. decreased oxidation. MetMb liposomes liberated free iron when they oxidised. DFO inhibited oxidation effectively, DTPA partial inhibition.
[61]	Soy PC, HSPC, HSPC+Chol chitosan, DC-chol, DPPA	Water	Cholesterol	Ascorbic acid	Type of PCs	Ferrous sulfate (0.24 M)	SPC, HSPC and HSPC+Chol liposomes protected iron from oxidation and presented high iron uptake by Caco-2 cells.
[62]	Soy PC	Citrate buffer (pH 6.6)	Lactalbumin	Raspberry, bilberry, lingonberry, black currant phenolics	Phenolic concentration (1.4, 4.2, and 8.4 μg/mL)	-	Lingonberry and bilberry phenolics were the most effective against phospholipid oxidation. Bilberry and raspberry phenolics exhibited best overall antioxidant activity towards protein oxidation.
[63]	Egg PC	Phosphate buffer (pH 6.8)	Cholesterol, ß-sitosterol, stigmasterol, bovine serum albumin	α-tocopherol	Type of sterols	-	Addition of sterols to liposomes decreased the oxidation. Replacing cholesterol with phytosterols on liposomes feasible.
[64]	Soy lecithin	67 mM phosphate buffer (pH 7.4)	-	BHT and various plant extracts	Type of plant extracts (200 μg/mL)	Ferric chloride (30 μM) and/or fructose (13 mM)	All extracts inhibited iron-fructose-phosphate-induced lipid oxidation in both lipid systems (lecithin liposomes or linoleic acid emulsions); *C. album* and *C. foliosum* extracts showed less inhibition than others.
[65]	Cod roe PL	MES solution (pH 3.3-7.0)	-	EDTA, oxalic acid, citric acid, casein	Buffer pH, metal chelators	Ferrous sulfate, ferrous chloride (7.5, 10, 15 μM)	Highest oxidation was at pH 4-5. pH >5.7 lowered solubility of Fe^{3+} and decreased oxidation rate. Liposome charge decreased with increasing pH. Chelators

							decreased Fe^{+2} mediated oxidation; EDTA~ casein > citric acid > oxalic acid
[66]	POPC, PLPC, POPA	Sodium chloride (146 mM)	Cholesterol	Urate	Presence of AAPH, copper chloride	Copper chloride (10 μM)	AAPH-induced peroxidation of cholesterol is slow and independent of peroxidation of PLPC. Cholesterol is not susceptible to copper-induced oxidation, but its inclusion in PLPC liposomes affects the peroxidation of PLPC, slowing down the initial stage of oxidation but promoting later stages. Addition of urate accelerates copper-induced peroxidation of PLPC in the absence of cholesterol, whereas in cholesterol-containing liposomes it inhibits PLPC oxidation
[67]	Soy PC	27.5 mM Citrate buffer (pH 6.6)	Bovine serum albumin, casein, lactalbumin	α-tocopherol, procyanidin anthocyanin, aglycons	Type of antioxidants	Copper acetate	Good correlation protein and lipid oxidation. All tested anthocyanin and other phenolic compounds inhibited both lipid and protein oxidation. Procyanidins B1 and B2 and ellagic acid were potentially better antioxidants than anthocyanin. Casein was the most stable protein in the liposome model and best inhibitor of liposome oxidation.
[68]	Egg PC	0.01 M phosphate buffer solution, 150 mM NaCl, phosphate buffer solution (pH 7.4)	Tween 80	Lutein, β-carotene, Lycopene, canthaxanthin	Type of carotenoids	-	Carotenoids exhibited antioxidant activities against lipid oxidation of liposomes ranging from the strongest to the weakest: lutein > β-carotene > lycopene > canthaxanthin.
[69]	Egg PC	50 mM phosphate buffer (pH 7.4)	Glucose	EGCG, ester derivatives of EGCG	Type of EGCG derivatives	-	The derivatives were more effective than EGCG against UV-induced liposome oxidation.
[70]	Soy PC	Succinic acid and succinic acid sodium salt (pH 4.7)	Bovine serum albumin	Ferulic acid, epicatechin, catechin, rutin, malvidin, caffeic acid, quercetin, propyl gallate	Type of phenolic compounds	Cupric acetate (3 μM)	Ferulic acid, epicatechin, and catechin provided better oxidative stability of liposomes than other phenolic compounds in the presence of bovine serum albumin.
[71]	Egg PC	2 mM NaCl solution (pH 6)		*Trans*-resveratrol, L-ascorbic acid, α-tocopherol, epicatechin, quercetin	Type of antioxidants	-	Trans-resveratrol was a better radical scavenger than vitamins E and C but similar to the flavonoids epicatechin and quercetin.
[37]	Cod roe PL, Herring roe PL, Soy PL, Bacterial PL	MES solution (pH 2-7)	TPP	-	Type of PLs, buffer pH	Ferrous sulfate, ferrous chloride (5-30 μM)	Oxidation was the highest for liposomes/emulsions at pH 4-5. Pro-oxidant effect of iron was reduced by less unsaturated phospholipids, specific emulsifier amounts, chloride anions, or xanthan gum.

[72]	DPPC	0.09 M citrate and 0.11 M formate (pH 3); 0.06 M citrate, 0.075 M formate, 0.11 M acetate (pH 4); 0.043 M citrate and 0.075 M acetate (pH 5)	Cholesterol	Citric acid, ascorbic acid (AA)	Copper ions, cholesterol, buffers	Copper sulfate (32 μM)	Oxidation of AA in a model aqueous liquid food (apple juice) was significantly slowed down by incorporation into liposomes. Oxidation of AA was reduced in copper-containing environment above pH >3. DPPC/chol liposomes were more stable at higher temperatures than DPPC.
[18]	DOPC, DPPC, DBPC, DOPE as stabilizers of emulsion	10 mM phosphate buffer (pH 7.0)	Tween 20	-	Effect of pH; unsaturation level of fatty acids; type of phosphate headgroup	-	DOPC showed higher oxidative stability at pH 7 than pH 3. PC with either oleic or palmitic acid were the most effective at inhibiting lipid oxidation. DOPC showed higher oxidative stability than DBPC at pH 7.
[12]	Egg PC	10 mM phosphate-citrate buffer (pH 6.8)	Cholesterol, Tween 20	-	Physical form of iron (free, or entrapped in liposomes); type of emulsifier in emulsion (Tween 20 or WPI)	Ferrous sulfate (61 mM)	Lipid oxidation was substantially higher in emulsions containing iron (either free, or encapsulated in liposomes) than in blank (iron-free) emulsions.
**Reference**	**Phospholipid type**	**Aqueous medium**	**Other components**	**Oxidation prevention**	**Factors**	**Pro-oxidant**	**Key findings**

Abbreviations: AA; ascorbic acid, AAPH; 2,2-azobis(2-amidinopropane)hydrochloride, Chol: cholesterol, DFO; Desferrioxamine, DHA; docosahexaenoic acid, DPPC; dipalmitoylphosphatidylcholine, DTPA; diethylenetriamine pentaacetic acid, EGCG; Epigallocatechin gallate, EDTA; Ethylenediaminetetraacetic acid, EPA; Eicosapentaenoic acid, HSPC; hydrogenated phatidylcholine, LOOH; lipid hydroperoxides, MES; 2-morpholi-nethanoesulfonic acid, PC; phosphatidylcholine, PL; phospholipids, PLPC; palmitoyllinoleoylphosphatidylcholine, POPC; 1-Palmitoyl 2- oleoyl-sn-glycero-3-phosphocholine, POPA; 1-palmitoyl-2-oleoyl-sn-glycero3-phosphate, ROS; reactive oxygen species, SA; Stearic acid, SOD; Superoxide dismutase, TPP; triphenylphosphine, WPI; Whey protein isolate.

Table S2. Fatty acid composition (μg/g) of phosphatidylcholine (PC).

Fatty acids	Content (μg/g)
Myristic acid (C 14:0)	6.23 ± 0.05
Palmitic acid (C16:0)	1388.09 ± 14.47
Stearic acid (C18:0)	703.57 ± 8.82
Arachidic acid (C20:0)	24.63 ± 0.74
Behenic acid (C22:0)	0.89 ±0.29
Lignoceric acid (C24:0)	ND
**Total SFA**	**2123.41**
Palmitoleic acid (C16:1*n-7*)	17.61 ± 0.79
Oleic acid (C18:1*n-9*)	878.13 ± 26.44
Erucic acid (C22:1*n-9*)	ND
**Total MUFA**	**895.74**
Linoleic acid (C18:2*n-6*)	212.10 ± 30.32
α-linolenic acid (C18:3*n-3*)	0.77 ± 0.3
**Total PUFA**	**212.88**
**Total FA**	**3232.03**

ND, not detected; SFA, saturated fatty acid; MUFA, monounsaturated fatty acid; PUFA, polyunsaturated fatty acid. esults are expressed as mean ± standard deviation of independent duplicates.

References

57. Mozuraityte, R.; Rustad, T.; Storrø, I. Pro-oxidant activity of Fe2+ in oxidation of cod phospholipids in liposomes. *Eur. J. Lipid Sci. Technol.* **2006**, *108*, 218–226.
58. Ohyashiki, Takao; Kadoya, Akinori ;Kushida, K. The Role of Fe 3+ on Fe 2+ -dependent lipid peroxidation in phospholipid liposomes. *Pharm. Soc. Japan* **2002**, *50*, 203–207.
59. Yamamoto, K.; Niki, E. Interaction of α-tocopherol with iron: antioxidant and prooxidant effects of α-tocopherol in the oxidation of lipids in aqueous dispersions in the presence of iron. *Biochim. Biophys. Acta - Lipids Lipid Metab.* **1988**, *958*, 19–23.
60. Min, B.; Nam, K.C.; Ahn, D.U. Catalytic mechanisms of metmyoglobin on the oxidation of lipids in phospholipid liposome model system. *Food Chem.* **2010**, *123*, 231–236.
61. Hermida, L.G.; Roig, A.; Bregni, C.; Sabés-Xamaní, M.; Barnadas-Rodríguez, R. Preparation and characterization of iron-containing liposomes: Their effect on soluble iron

uptake by Caco-2 cells. *J. Liposome Res.* **2011**, *21*, 203–212.
62. Viljanen, K.; Kylli, P.; Kivikari, R.; Heinonen, M. Inhibition of protein and lipid oxidation in liposomes by berry phenolics. *J. Agric. Food Chem.* **2004**, *52*, 7419–7424.
63. Chan, Y.H.; Chen, B.H.; Chiu, C.P.; Lu, Y.F. The influence of phytosterols on the encapsulation efficiency of cholesterol liposomes. *Int. J. Food Sci. Technol.* **2004**, *39*, 985–995.
64. Mavi, A.; Lawrence, G.D.; Kordali, S.; Yildirim, A. Inhibition of iron-fructose-phosphate-induced lipid peroxidation in lecithin liposome and linoleic acid emulsion systems by some edible plants. *J. Food Biochem.* **2011**, *35*, 833–844.
65. Mozuraityte, R.; Kristinova, V.; Rustad, T.; Storrø, I. The role of iron in peroxidation of PUFA: Effect of pH and chelators. *Eur. J. Lipid Sci. Technol.* **2016**, *118*, 658–668.
66. Schnitzer, E.; Pinchuk, I.; Bor, A.; Leikin-Frenkel, A.; Lichtenberg, D. Oxidation of liposomal cholesterol and its effect on phospholipid peroxidation. *Chem. Phys. Lipids* **2007**, *146*, 43–53.
67. Viljanen, K.; Kivikari, R.; Heinonen, M. Protein-lipid interactions during liposome oxidation with aAdded anthocyanin and other phenolic compounds. *J. Agric. Food Chem.* **2004**, *52*, 1104–1111.
68. Tan, C.; Xue, J.; Abbas, S.; Feng, B.; Zhang, X.; Xia, S. Liposome as a delivery system for carotenoids: Comparative antioxidant activity of carotenoids as measured by ferric reducing antioxidant power, DPPH assay and lipid peroxidation. *J. Agric. Food Chem.* **2014**, *62*, 6726–6735.
69. Zhong, Y.; Shahidi, F. Lipophilised epigallocatechin gallate (EGCG) derivatives and their antioxidant potential in food and biological systems. *Food Chem.* **2012**, *131*, 22–30.
70. Heinonen, M.; Rein, D.; Satué-Gracia, M.T.; Huang, S.W.; German, J.B.; Frankel, E.N. Effect of protein on the antioxidant activity of phenolic compounds in a lecithin-liposome oxidation system. *J. Agric. Food Chem.* **1998**, *46*, 917–922.
71. Stojanović, S.; Sprinz, H.; Brede, O. Efficiency and mechanism of the antioxidant action of trans-resveratrol and its analogues in the radical liposome oxidation. *Arch. Biochem. Biophys.* **2001**, *391*, 79–89.
72. Wechtersbach, L.; Poklar Ulrih, N.; Cigić, B. Liposomal stabilization of ascorbic acid in model systems and in food matrices. *LWT - Food Sci. Technol.* **2012**, *45*, 43–49.
